# Supplementary material for: Characteristics of Buffalo Farming Systems in Turkey Based on a Multivariate Aggregation of Indicators: A Survey Study
Source: Animals (Basel). 2022 Nov 7;12(21):3056. doi: 10.3390/ani12213056 (PMC9658953; doi:10.3390/ani12213056)
Supplement: Supplementary file 1 [file animals-12-03056-s001.zip › animals-1985434-supplementary.pdf]

Table S1. Equations and references that were used to calculate the indicators.

Indicator	Calculation	References
Herd Health Information		
Culling rate	Number of female buffaloes leaving the herd / Total number of female buffaloes	[61]
Economic Information		
Gross production value	Dairy income + live animal selling income + subsidies + productive asset value increase	[62]
Dairy income	Income generated from selling milk + yoghourt + kaymak. The calculation was performed as production quantity x corresponding price.	[62]
Productive asset value increase	[Herd value at the end of the production year + sold live animal value + slaughtered animal value] – [Herd value in the beginning of the production year + purchased animal value]	[62]
Gross profit	Gross production value – variable costs	[62]
Variable costs	Feed cost + temporary labor cost + health cost + electricity and water cost + annual loan interest	[62]
Annual work unit	The annual work unit of a worker was accepted as 1800 hours / year.	[63]

Table S2. General characteristics of the farms corresponding to their location.

Attributes	ISTANBUL		KOCAELI		BALIKESIR	
	N	Relative Frequency	N	Relative Frequency	N	Relative Frequency
Grazing Information						
Own land grazing	1	4.0	16	94.1		
Meadow grazing	14	56.0	1	5.9	10	100.
Forest grazing	3	12.0				
Mixed Grazing (Meadow + Forest)	7	28.0				
Animal Nutrition information-Summer						
Concentrate suppliers	23	92.0	10	58.8	7	70.0
Wheat suppliers	7	28.0	1	5.9	0	0.0
Wheat shorts suppliers	7	28.0	0	0.0	0	0.0
Wheat bran suppliers	3	12.0	2	11.8	10	100.0
Barley suppliers	14	56.0	3	17.6	7	70.0
Beet pulp suppliers	1	4.0	0	0.0	0	0.0
Red dog suppliers	16	64.0	0	0.0	0	0.0
Cotton seed suppliers	0	0.0	0	0.0	7	70.0

Straw (barley or wheat) suppliers	22	88.0	9	52.9	8	80.0
Alfalfa hay suppliers	17	68.0	11	64.7	9	90.0
Corn silage suppliers	5	20.0	12	70.6	8	80.0
Animal Nutrition information-Winter						
Concentrate suppliers	23	92.0	13	76.5	6	60.0
Wheat suppliers	4	16.0	4	23.5	0	0.0
Wheat shorts suppliers	12	48.0	0	0.0	0	0.0
Wheat bran suppliers	1	4.0	4	23.5	10	100.0
Barley suppliers	14	56.0	10	58.8	4	40.0
Beet pulp suppliers	3	12.0	1	5.9	0	0.0
Red dog suppliers	16	64.0	0	0.0	0	0.0
Cotton seed suppliers	0	0.0	0	0.0	4	40.0
Straw (Barley or wheat) suppliers	25	100.0	15	88.2	7	70.0
Alfalfa hay suppliers	22	88.0	14	82.4	9	
Corn silage suppliers	11	44.0	16	94.1	9	
Breed information						
Anatolian Buffalo	25	100.0	17	100.0	10	100.0
Milking Information						
Milking technique						
Automatic milking	14	56.0	11	64.7	5	50.0
Hand milking	11	44.0	6	35.3	5	50.0
Milking frequency						
Twice a day	25	100.0	17	100.0	10	100.0
Reproductive information						
Insemination technique						
Natural insemination	25	100.0	17	100.0	10	100.0
Health Information						
Calves Diarrhoea	3	12.0	2	11.8	2	20.0
Mastitis	4	16.0	1	5.9	0	0.0
No case report	18	72.0	14	82.3	8	80.0
Farms reported abortion						
Yes	10	40.0	9	52.9	4	40.0

Table S3. Percentage of farms with 0 values corresponds to each indicator.

Indicators	Number	Relative Frequency
**Land use information**		
Land used for grain production, ha	21	40
Land used for roughage production, ha	8	15
Land used for roughage and grain production per breeding buffalo, ha	6	21
**Herd information**		
Breeding buffalo / Buffalo bull	3	6
**Animal nutrition information**		
Concentrate-to-roughage ratio in spring-summer	11	21
Silage in diet, winter, %	16	31
Silage in diet, summer, %	26	50
**Herd health information**		
Culling rate	19	37
**Economic performance information**		
Milk income, US$	9	17
Yogurt income, US$	38	73
Milk price per kg, US$	9	17
Yogurt price per kg, US$	38	73
Kaymak price per kg, US$	44	85
Purchased feed cost / Total feed cost	2	4
Fattening calves' income / Dairy income, %	30	58
**Labor information**		
Family labour / Total labour, AWU	7	13
Paid labour / Total labour, AWU	8	58

Technical and Economic Analysis of the Buffalo Farms in Marmara Region

Farm No : Date: …../…../…..

Time:

Season: (1) Fall-winter

(2) Spring-summer

Province:

Farmer's NAME: Phone number:

A. Farmer information:

A1. Age: ………… and Gender: (male) (female)

A2. Number of households: (1) (2) (3) (4) (5) (6) (other ………………….)

A3. Experience in buffalo husbandry: ………………………………….. (month / year)

A4. Education status: (…) Literate, (…) Primary school, (…) Secondary school, (…) High school, (…) Vocational school, (…) University, (…) Master's degree-PhD, (…) Illiterate

A5. Monthly household income level except buffalo husbandry (₺): …………………

A6. Is the farm property belonging to you? (…) yes, (…) no

If rent, how much is the monthly rent?…………….

B. Land use:

B1. Agricultural land (ha):……..

B1a. Cultivated pasture land (ha):……………

B1b. Cultivated roughage production land (ha):….

B1c. Cultivated grain production land (ha):……..

B2. Natural pasture land (ha):…….

B2a. Scrub land (ha):……..

B2b. Meadow land (ha):………..

B2c. Forest land(ha):………

B3. Farm area (ha):…….

C. Herd structure:

C1. Breeds of farming buffaloes:

i) ……….. number of female Anatolian Buf.<br><br>i) ……….. number of male Anatolian Buf.	ii) ……. number of female Italian Buf.<br><br>ii) ……. number of male Italian Buf.	iii) ……. . number of cross breed female Buf.<br><br>iii)………. number of cross breed male Buf.

C2. Herd size: ……. number of breeding buffaloes (all breeds); ………. number of buffaloes giving first birth; ………. number of buffaloes giving 2 and more birth

C3. Number of buffalo calves: ………. number of female buffalo calves ; …….. number of male buffalo calves

Additional notes:

D. Animal nutrition and pasture use:

**A:** only barn; **M:** only pasture; **X:** pasture+barn	JAN	FEB	MAR	APR	MAY	JUN	JUL	AUG	SEP	OCT	NOV	DEC
**D1.** Female breeding herd:												
**D2.** Male buffalo calves:												
**D3.** Female buffalo calves:												
**D4.** Buffalo bull:												

D5a. What is the amount of feed given to animals during the corresponding period?

	**LACTATING BUFFALO (N):**	**DRY BUFFALO (N):**	**YOUNG BUFFALO(N):**	**BUFFALO BULL (N):**
CONCENTRATE FEED:				
FATTENING FEED:				
BARLEY:				
WHEAT:				
WHEAT SHORT:				
WHEAT BRAN:				
RED DOG:				
COTTON SEED:				
CORN:				
BEER PULP				
**ROUGHAGE**				
ALFALFA-HAY				
STRAW:				
SILAGE:				

D7. Total amount of roughage offer (kg):…………………….

D8. Total amount of concentrate offer (kg):……………

D9a. Pasture enter and return hours in winter season …………..- …………

D9b. Pasture enter and return hours in spring season: …………-…………..

D9c. Pasture enter and return hours in summer season:………..-…………

D9d. Pasture enter and return hours in fall season: ……….-………………..

D10. Weaning age of buffalo calves (day): ……….

Additional notes:

E. Health, breeding, selection ve debugging information:

E1. Culling rate: ……….

E2. Reported diseases:…………………………………………………………………………….

E3a. Have you experienced abortion this year so far? (…) yes, (…) no

E3b. Which gestation period has abortion occurred? …………………………………………..

E4. Duration of use in breeding (year): buffalo bulls: ………., female buffaloes: ……….

E5. Age of first use in breeding(month): female breeding buffaloes: …………, male breeding buffaloes: …….

E6a. Number of dead calves:……….

E6b. Number of dead buffalo:………….

Additional notes:

F. Animal production

S: Start; F: Finish	Jan	Feb	Mar	Apr	May	Jun	Jul	Aug	Sep	Oct	Nov	Dec
**Lactating buffalo (N)**												
**Produced milk (L)**												

F2. Milking technique: (……) by hand, (……) by machine, Other: ………………..

F3. Daily milking time: (…) Only morning, (…) Only evening, (…) Morning + Evening, (…) Other…………………………………………………………………

F4. Milker: (…) Shepherd, (…) Another milker, (…) Man in family, (…)Woman in family, (…) Other………………………………………

F5. Days in milk: ……….

Additional notes:

G. Economic performance:

G1. Which enterprise your feed is being purchased?..

BUFFALO MILK PRODUCTION COSTS	
**Cost elements**	**Purchase or production cost**
Concentrate<br>Fattening feed<br>Barley<br>Wheat<br>Wheat short<br>Wheat bran<br>Red dog<br>Cotton seed<br>Corn<br>Beet pulp	
Alfalfa hay<br>Straw<br>Silage	
Cost of milk given to the calves	
Breeding female animal cost	
Breeding male animal cost	
Temporary labour cost	
Temporary labour duration (month):	
Water and energy cost	
Health cost (Veterinary, vaccine, medicine, artificial insemination)	
Bedding cost	
Loan interest (if any)	
Other costs	

Additional notes:

YOGHURT / KAYMAK PRODUCION COSTS	
**Cost elements:**	**Costs (TL/year)**
Cow milk	
Labour	
Energy (Electricity or gas)	
Yoghurt/Kaymak pot	
Water	

Used buffalo milk amount for producing 1 kg yoghurt/kaymak:........

Used cow milk amount for use producing 1 kg yoghurt:/kaymak:........

G2. Market supply situations of income elements

**Product**	**Amount of prod.**	**Used in family**	**Amount of sold**	**Price (TL/kg)**
Milk (kg)				
Manure (kg)				
Kaymak (kg)				
Yoghurt (kg)				
Livestock (N)				

G3. Where/Who do you sell the animal products, what percentage? (..., ...%) Union, (..., ... %) Cooperative, (...,....%) Dairy company, (...,....%) Regional dairy, (...,....%) Farmer market, (...,....%) other

G4a. Do you benefit from the livestock supports? (...) yes, (...) no

G4b. What supports do you obtain? (...) 4 months and above calve support, (...) Breed registry, (...) milk support, (...) cultivated feed crops, (...) National breeding buffalo project[1], (...) Breeding buffalo calve support.

G5. Financial values of animals at the beginning and end of the year

	January 2018		Between January 2018 and December 2018						December 2018	
			Purchased		Sold		Dead			
	Number	Price	Number	Price	Number	Price	Number	Price	Number	Price
Female buffalo										
Buffalo bull										
Male buffalo calve (0-6 month)										
Female buffalo calve (0-6 month)										
Female breeding buffalo (6 month- 3 age)										
Male breeding buffalo (6 month- 3 age)										

Additional notes:

H. Labour:

H1. How many members of your family actively attend to buffalo husbandry? (...) 1, ( ) 2,( ) 3,(....) 4, (.....) 5, (....) more than 5

H2a. How many hours do a family member work per day? ....

H2b. How many days do a family member work per week?........

H3a. Do you use paid labour? (...) yes, (...) no

H3b. What is the number of paid labours? ……….

H3c. What is the monthly cost for paid labour?

H3d. How many hours does your labour work daily? From…., until……….

H3e. How many days does your labour work weekly?........

H4a. Number of paid labour younger than 40 years old……….

H4b. Number of family members younger than 40 years old and actively attending to buffalo husbandry:

I. Investments in buildings and equipment:

**Item**	**Yes (1), No (0)**
Milking unit	
Milk cooling tank	
Generator	
Feed crushing unit	
Feed mixing unit	
Buffalo barn	
Courtyard	
Concentrate forage storage	
Roughage storage	
Worker office/lodging	
**Total score**	

J. Farmer profile:

J1. Did you attend a training on animal husbandry? (…) yes, (…) no

If you attend, duration(day): ……….

J2a. Do you receive a technical advice? (…) yes, (…) no

J2b. If you are receiving, please indicate the field.

**Field**	**Yes (1), No (0)**
Health	
Reproduction	
Animal nutrition	
Pasture-cultivated land	
**Total score**	

J3a. Are you a member of a cooperative, union or association related to animal husbandry?

(…) yes, (…) no

J3b. If you are a member, please indicate which cooperative, union or association is it?……….
